# Insect Repellents: Modulators of Mosquito Odorant Receptor Activity

**DOI:** 10.1371/journal.pone.0012138

**Published:** 2010-08-11

**Authors:** Jonathan D. Bohbot, Joseph C. Dickens

**Affiliations:** Invasive Insect Biocontrol and Behavior Laboratory, Henry A. Wallace Beltsville Agricultural Research Center, Plant Sciences Institute, Agricultural Research Service, United States Department of Agriculture, Beltsville, Maryland, United States of America; University of California Los Angeles, United States of America

## Abstract

**Background:**

DEET, 2-undecanone (2-U), IR3535 and Picaridin are widely used as insect repellents to prevent interactions between humans and many arthropods including mosquitoes. Their molecular action has only recently been studied, yielding seemingly contradictory theories including odorant-dependent inhibitory and odorant-independent excitatory activities on insect olfactory sensory neurons (OSNs) and odorant receptor proteins (ORs).

**Methodology/Principal Findings:**

Here we characterize the action of these repellents on two *Aedes aegypti* ORs, AaOR2 and AaOR8, individually co-expressed with the common co-receptor AaOR7 in *Xenopus* oocytes; these ORs are respectively activated by the odors indole (AaOR2) and (*R*)-(−)-1-octen3-ol (AaOR8), odorants used to locate oviposition sites and host animals. In the absence of odorants, DEET activates AaOR2 but not AaOR8, while 2-U activates AaOR8 but not AaOR2; IR3535 and Picaridin do not activate these ORs. In the presence of odors, DEET strongly inhibits AaOR8 but not AaOR2, while 2-U strongly inhibits AaOR2 but not AaOR8; IR3535 and Picaridin strongly inhibit both ORs.

**Conclusions/Significance:**

These data demonstrate that repellents can act as olfactory agonists or antagonists, thus modulating OR activity, bringing concordance to conflicting models.

## Introduction

**Figure 1:**
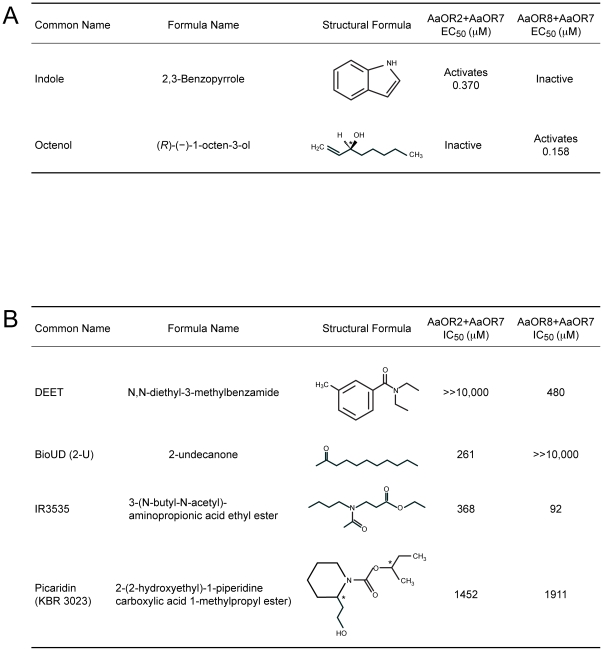
Compounds identification and repellent effectiveness on AaORs. (A) Structural formulas of odorants and (B) half maximal inhibitory concentrations (IC_50_) of insect repellents. Asterisks indicate chiral centers.

The exact modes of action and molecular targets of the active ingredients found in insect repellents are poorly understood. Addressing this gap in our knowledge has become an urgent matter in order to understand how to improve the effectiveness of repellents and to develop a novel generation of olfactory disruptive compounds. Currently, most insect repellent products include the active ingredients N,N-diethyl-3-methylbenzamide (DEET), Insect Repellent 3535 (IR3535), and more recently Picaridin and 2-undecanone (2-U) (Fig. 1). In the current study, we investigate the molecular action of these repellents on two isolated odorant receptors (ORs) of the yellow fever mosquito *Aedes aegypti*.

Since its development by the military and the USDA in 1946 [1], the synthetic compound DEET has been the gold standard of insect repellents and has been used by both military and civilian populations alike. In addition, DEET may directly target insect acetylcholinesterases [2], mosquito ORs [3], [4] and it may chemically sequester a mosquito attractant [5]. In practice, DEET reduces bites from mosquitoes, ticks and other blood feeding arthropods [6] which may vector pathogens that cause diseases including malaria, yellow fever, West Nile virus, Lyme disease and dengue. IR3535 and Picaridin (also known as KBR 3023, Icaridine, and Bayrepel) were developed in the 1970s and 1990s [7], and are also of synthetic origin. 2-U is a naturally occurring compound produced by the glandular trichomes of wild tomato plants as part of a plant defense mechanism against herbivorous insects [8] and was shown to have mosquito repellent properties at high concentrations [9]. In 2007, 2-U was incorporated in the insect repellent BioUD (HOMS LLC, Clayton, NC) for its repellent properties on various arthropods including mosquitoes [10] and ticks [11]. Additionally, 2-U was identified from Bermuda grass infusions and was shown to elicit electrophysiological responses from *Culex* antennae [9].

DEET, 2-U, IR3535 and Picaridin are broad spectrum arthropod repellents and exhibit similar efficacies [6] when used in large amounts. Commercial formulations are characterized by high concentrations of active ingredients, e.g., DEET formulations typically contain 5% to 100% DEET [12] while 2-U, IR3535 and Picaridin formulations range from 5% to 20% [6], [10]. There is evidence that the repellent and deterrent activities of DEET and Picaridin involve olfactory sensing in mosquitoes [13], [14], [15] and ticks [16] via their interactions with ORs [3], [4].

Insect ORs belong to a highly divergent gene superfamily, with little sequence similarity at the amino-acid level both within and between species. It is therefore important to recognize that these repellents may carry out their effects on arthropod behaviors via widely differing actions. Recent studies have characterized the mode of action of DEET on isolated ORs [3], [4] and olfactory sensory neurons (OSNs) of mosquitoes [5]. In one study, DEET was shown to inhibit the odorant-dependent activity of specific ORs [4]. In other studies, DEET was shown to directly activate a larval OR [3] sensitive to fenchone and stimulate a specific OSN known to be responsive to repellents [5] in adults. These opposite activities are consistent with previous behavioral and physiological observations: DEET has been shown to reverse the effect of otherwise attractive odorants (i.e. induce a repellent effect) in ticks [16], moths [17] and mosquitoes [7], [18]; DEET presented alone has been shown to act as either a repellent [5] or an attractant [19] in mosquitoes. In *Aedes aegypti*, DEET's inhibitory activity against attractive odorants was shown to be a result of a reduction in the sensitivity threshold of the OSNs to lactic acid [19], [20] or to the oviposition attractant ethyl propionate [21]. Similar to DEET, 2-U may have multiple effects on *Ae. aegypti'*s behavior: acting both as an attractant [22] and a repellent at high concentrations [10]. In female *Culex quinquefasciatus*, 2-U was shown to activate antennal OSNs responding to carboxylic acids and monoterpenes [23].

In our study, we investigate the action of 4 insect repellents on the activities of two *Ae. aegypti* ORs, AaOR2 and AaOR8, respectively, expressed in *Xenopus* oocytes together with AaOR7. Mosquito ORs govern odor specificity, but form obligate hetero-complexes with the common co-receptor OR7 [24], [25], [26], [27], [28], [29]; OR7 is the ortholog of *Drosophila melanogaster* OR83b [30], [31]. Ditzen et al. (2008) previously characterized DEET interactions with *Anopheles gambiae* ORs co-expressed with AgOR7; activation of AgOR2 by 2-methyl phenol and AgOR8 by racemic 1-octen-3-ol was differentially inhibited by DEET suggesting that DEET selectively inhibited the different odor-specific subunits (OR2 and OR8) rather than the common co-receptor (OR7) [4]. AgOR2 was more recently shown to be 100-fold more sensitive to the oviposition attractant indole relative to 2-methyl phenol [32]. We recently showed that *Ae. aegypti* AaOR8, the ortholog of AgOR8, is sensitive to 1-octen-3-ol and enantioselective, 100× more sensitive to (*R*)-(−)-1-octen-3-ol (henceforth termed octenol in following text and figures) than to (*S*)-(+)-1-octen-3-ol [33]. We have also shown that *Ae. aegypti* AaOR2, the ortholog of AgOR2 [34] and CxOR2 [35], exhibits similar sensitivity to indole (Fig. S1).

Here we report the influence of the repellents DEET, 2-U, IR3535 and Picaridin on the responses of AaOR2 and AaOR8 to their respective agonists indole and octenol. AaOR2 and AaOR8 were expressed in *Xenopus* oocytes along with their hetero partner AaOR7, and activities were characterized using two electrode voltage-clamp electrophysiology. Our results provide further evidence that DEET interacts with mosquito ORs. More importantly, they clarify previous observations that DEET and other insect repellents can have multiple effects on different ORs, which should interfere with mosquito OSNs, leading to behavioral disruption and reduced vectorial capacity.

## Results

### Stimulatory effects of odorants alone on AaOR2 and AaOR8

We first characterized the stability of our OR-*Xenopus* expression system to repeated odor stimulations (Fig. S2). AaOR2 and AaOR8 were individually expressed in *Xenopus* oocytes along with AaOR7, as in all subsequent studies, and repeatedly stimulated with 10^−7^ M indole (OR2) or octenol (OR8) under otherwise identical conditions (Fig. S2A). We chose concentrations of agonist in the lower portion of the dose-response dynamic ranges for AaOR2 (Fig. S1) and AaOR8 [33] in order to minimize signal desensitization, which tends to increase at higher concentrations. For both ORs, repeated odorant stimulations induced only a minor linear reduction of odorant-evoked inward currents (Fig. S2B). Between stimulations, oocytes were allowed to return to their membrane resting potential (recovery time) by washing out the odorants using pure Ringer's solution. Recovery times for AaOR2 (1.70±0.07 min, n = 40) and AaOR8 (1.78±0.05 min, n = 80) did not vary significantly across stimulations (P>0.05; *t*-test). In general, AaOR8 injected oocytes exhibited higher inward current responses than AaOR2 preparations (Fig. S2A). These results indicated that the preparations should be stable throughout the time courses of subsequent studies.

### Stimulatory effects of repellents alone on AaOR2 and AaOR8

**Figure 2:**
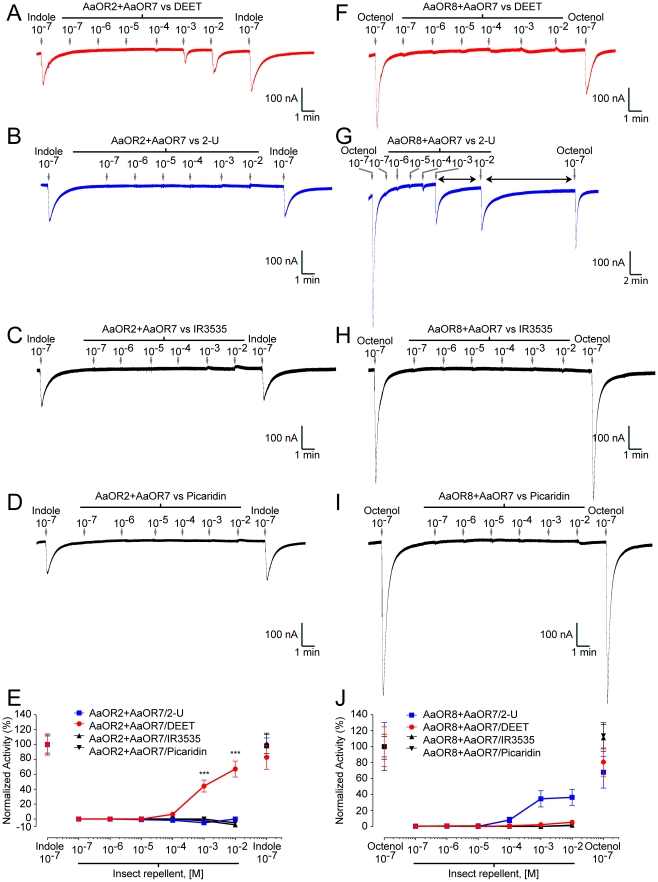
DEET and 2-undecanone alone, selectively activate AaORs. Response traces and concentration-response curves of AaOR2+AaOR7 and AaOR8+AaOR7 exposed to DEET (red), 2-U (blue), IR3535 and Picaridin are recorded in nano-ampere (nA). (A) DEET activates AaOR2+AaOR7. (B) (C) and (D) 2-U, IR3535 and Picaridin do not activate AaOR2+AaOR7. (E) The concentration-response plots of AaOR2+AaOR7 to increasing amounts of repellents. (F) (H) and (I) DEET, IR3535 and Picaridin do not activate AaOR8+AaOR7. (G) 2-U activates AaOR8+AaOR7. Horizontal arrows indicate prolonged recovery times. Inward currents are shown as downward deflections. (J) The concentration-response plots of AaOR8+AaOR7 to increasing amounts of repellents. Odorant concentrations were plotted on a logarithmic scale. All concentrations are in molarity. Each point represents the mean current response and vertical error bars are s.e.m. n = 5 oocytes for each treatment.

We next characterized the OR response to repellents alone, in the absence of applied odorants (Fig. 2). AaOR2 was activated by DEET in a concentration-dependent manner, producing minimal inward currents at 10^−4^ M that increased with DEET concentrations up to 10^−2^ M (Fig. 2A and 2E); AaOR2 showed no response to 2-U, IR3535 or Picaridin at concentrations up to 10^−2^ M (Fig. 2B–D). In contrast, AaOR8 was activated by 2-U in a concentration-dependent manner, producing minimal inward currents at 10^−4^ M that increased with 2-U concentration up to 10^−2^ M (Fig. 2G and 2J); AaOR8 showed no response to DEET, IR3535 or Picaridin at concentrations up to 10^−2^ M (Fig. 2F, 2H and 2I). None of the repellents elicited currents in oocytes in the absence of ORs (water injected controls) (Fig. S3). These results show that AaOR2 and AaOR8 are differentially sensitive (activated) to DEET and 2-U in the absence of applied odorant.

### Inhibitory effects of DEET on AaOR2 and AaOR8 responses to odorants

**Figure 3:**
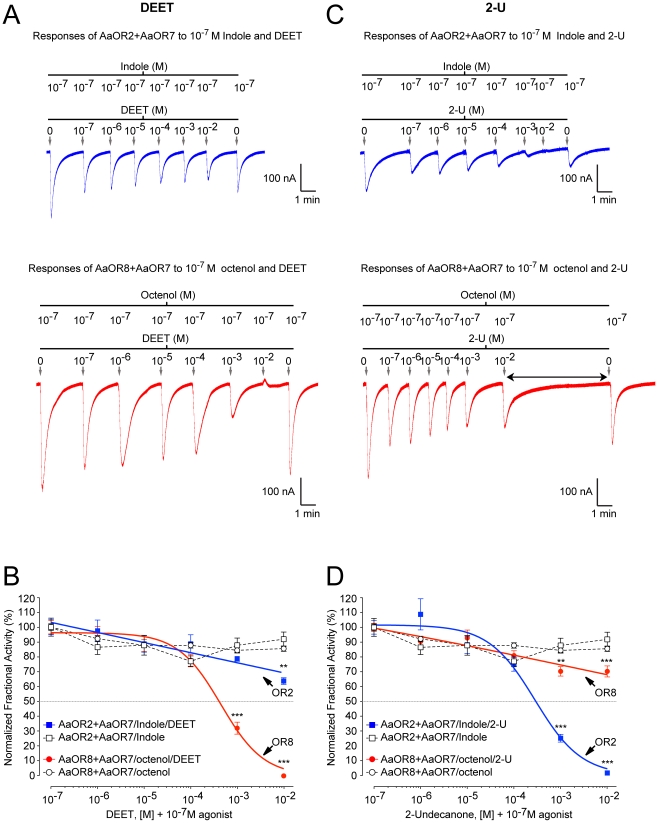
DEET and 2-undecanone selectively inhibit odorant-induced responses of AaORs. The concentration-response plots of AaOR2+AaOR7 and AaOR8+AaOR7 to repeated exposures of 10^−7^ M indole (open squares) and 10^−7^ M octenol [(*R*)-(−)-1-octen3-ol] (open circles) were duplicated in each panel for comparative purposes. (A) Response traces of AaOR2+AaOR7 (blue) and AaOR8+AaOR7 (red) to 10^−7^ M agonist alone and in combination with increasing concentrations of DEET (10^−7^ M to 10^−2^ M) are recorded in nano-ampere (nA). (B) Concentration-response plots of AaOR2+AaOR7 (solid blue squares) and AaOR8+AaOR7 (solid red circles) to 10^−7^ M indole and 10^−7^ M octenol in the presence of increasing amounts of DEET. (C) Response traces of AaOR2+AaOR7 (blue) and AaOR8+AaOR7 (red) to 10^−7^ M agonist alone and in combination with increasing concentrations of 2-undecanone (2-U) (10^−7^ M to 10^−2^ M) are recorded in nano-ampere (nA). Horizontal arrow indicates prolonged recovery time. (D) Concentration-response plots of AaOR2+AaOR7 (solid blue squares) and AaOR8+AaOR7 (solid red circles) to 10^−7^ M indole and 10^−7^ M octenol in the presence of increasing amounts of 2-U. Inward currents are shown as downward deflections. Odorant concentrations were plotted on a logarithmic scale. Each point represents the mean current response; error bars are s.e.m. n = 5–6 oocytes for each treatment. Treatments with high DEET concentrations (10^−3^ M and 10^−2^ M) and 2-U (10^−3^ M and 10^−2^ M) differed significantly from the no-repellent controls (two-way ANOVA, Bonferroni posttests, **: *P*<0.01; ***: *P*<0.001). Vertical and horizontal scale bars represent 100 nA and 1 min, respectively.

AaOR2 and AaOR8 were exposed to a range of DEET concentrations (10^−7^ M−10^−2^ M) in presence of their respective odorants indole and octenol (both at 10^−7^ M) (Fig. 3A and 3B). DEET inhibited the response to odorants for both ORs (Fig. 3A), however, at different sensitivities. AaOR8 response to octenol was strongly and significantly inhibited by DEET at 10^−3^ M (activity reduced to 30%); activity was entirely abolished at 10^−2^ M (Fig 3B). In contrast, AaOR2 response to indole was only slightly inhibited by DEET; inhibition did correlate (r^2^ = 0.905) with DEET concentration, but was only significant at 10^−2^ M DEET (ANOVA; P<0.01, Bonferroni posttest) (Fig. 3A and 3B). In all cases, the inhibitory effects of DEET on AaOR8 and AaOR2 were reversible using a final exposure of 10^−7^ M octenol or indole, respectively (Fig. 3A).

### Similar amounts of octenol are extracted from physiological solutions with or without DEET

**Figure 4:**
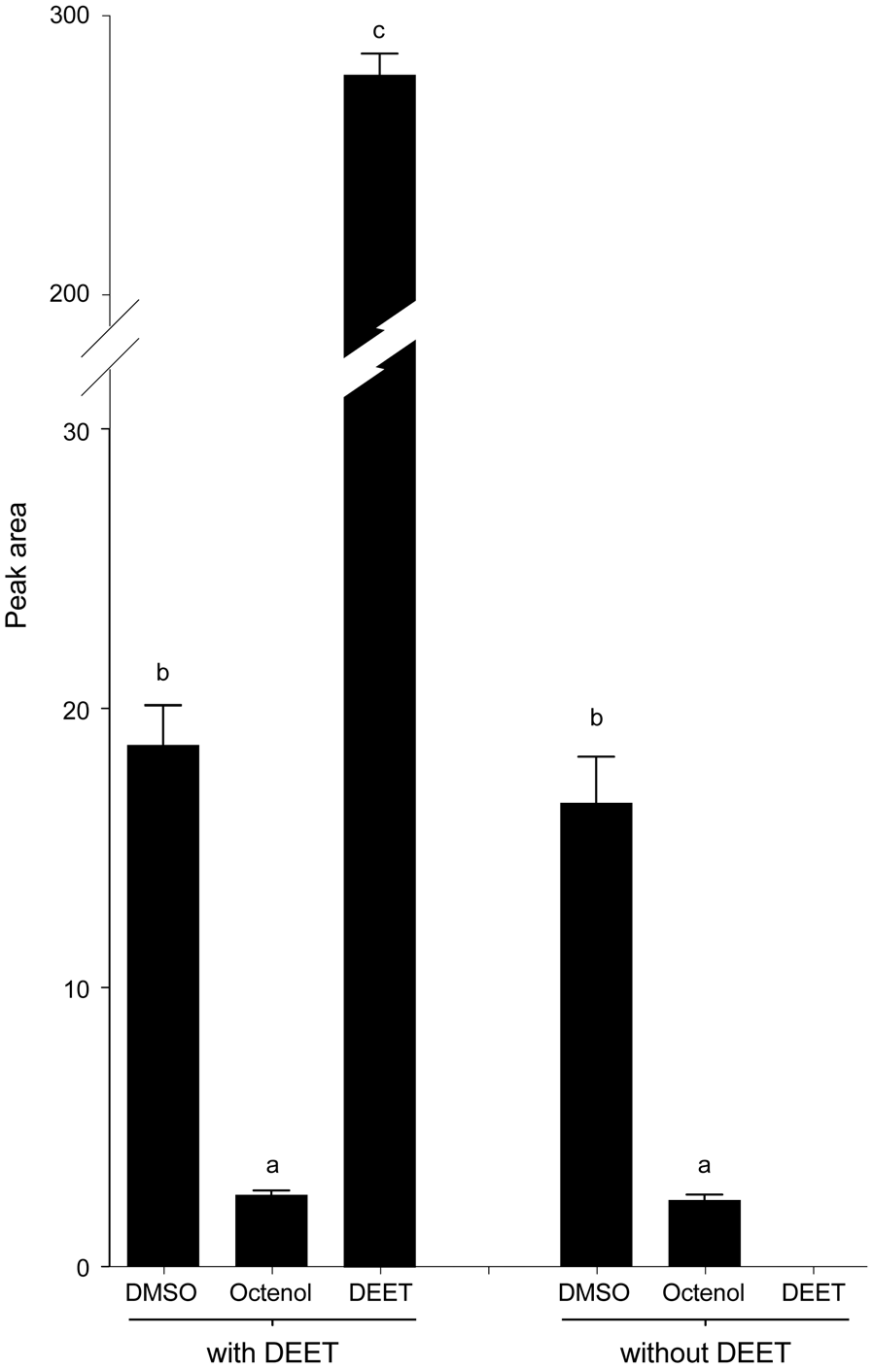
Octenol is not modified by DEET in solution. The presence of DEET (10^−3^ M) does not affect the mean amount of octenol [(*R*)-(−)-1-octen-3-ol)] (10^−5^ M) in physiological solution. Vertical bars represent s.e.m. (n = 5). Note broken x-axis for data representation. Same letters above histograms indicate non significant differences (*P*>0.05, ANOVA test with Tukey posttest).

One possible explanation for the inhibitory effects observed for DEET on responses of AaOR8 to octenol is that DEET might reduce the amount of ligand available for delivery to the receptor. This reduction in the amount of the proper ligand might be accomplished by diminishing the amount of octenol present in the solution due to the reactivity of the amide and carbonyl moieties present in the DEET molecule with octenol. Extracts of physiological solutions containing DMSO and octenol with or without DEET revealed nearly identical quantities of both compounds (Fig. 4). Thus, octenol did not appear to be reacting with DEET as quantities of octenol remained the same and no additional compounds were present in significant amounts in either of the solutions.

### Inhibitory effects of 2-U on AaOR2 and AaOR8 responses to odorants

AaOR2 and AaOR8 were exposed to a range of 2-U concentrations (10^−7^ M−10^−2^ M) in the presence of their respective odorants indole and octenol (both at 10^−7^ M) (Fig. 3C and 3D). 2-U inhibited responses to odorants for both ORs (Fig. 3C), albeit at different sensitivities. AaOR2 response to indole was strongly and significantly inhibited by 2-U at 10^−3^ M (activity reduced to 30%); activity was entirely abolished at 10^−2^ M (Fig 3D). In contrast, AaOR8 response to octenol was slightly inhibited by 2-U but was only significant at both 10^−3^ M and 10^−2^ M (ANOVA; P<0.01, Bonferroni posttest) (Fig 3D). The main effect of 2-U on AaOR8 was to prolong signal recovery, an effect only detected at the highest 2-U concentrations (Fig. 3C, Fig. S4). In all cases, the inhibitory effects of 2-U on AaOR8 and AaOR2 were reversible using a final exposure of 10^−7^ M octenol or indole, respectively (Fig. 3C).

### Inhibitory effects of IR3535 and Picaridin on AaOR2 and AaOR8 responses to odorants

**Figure 5:**
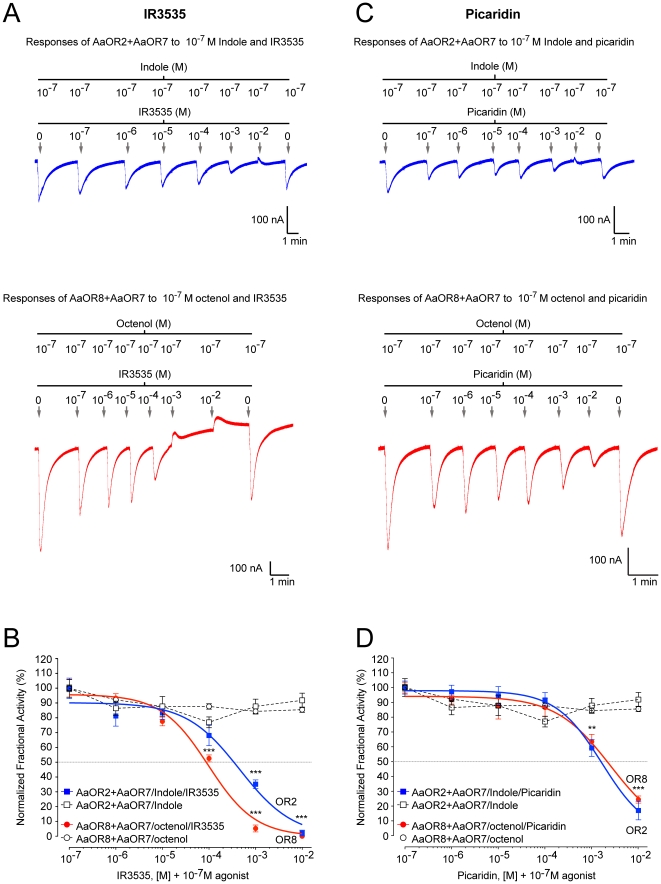
IR3535 and Picaridin inhibit odorant-induced responses of AaORs. The concentration-response plots of AaOR2+AaOR7 and AaOR8+AaOR7 to repeated exposures of 10^−7^ M indole (open squares) and 10^−7^ M octenol [(*R*)-1-octen3-ol)] (open circles) were duplicated in each panel for comparative purposes. (A) Response traces of AaOR2+AaOR7 (blue) and AaOR8+AaOR7 (red) to 10^−7^ M agonist alone and in combination with increasing concentrations of IR3535 (10^−7^ M to 10^−2^ M) are recorded in nano-ampere (nA). (B) Concentration-response plots of AaOR2+AaOR7 (solid blue squares) and AaOR8+AaOR7 (solid red circles) to 10^−7^ M indole and 10^−7^ M octenol in the presence of increasing amounts of IR3535. (C) Response traces of AaOR2+AaOR7 (blue) and AaOR8+AaOR7 (red) to 10^−7^ M agonist alone and in combination with increasing concentrations of Picaridin (10^−7^ M to 10^−2^ M) are recorded in nano-ampere (nA). (D) Concentration-response plots of AaOR2+AaOR7 (solid blue squares) and AaOR8+AaOR7 (solid red circles) to 10^−7^ M indole and 10^−7^ M octenol in the presence of increasing amounts of Picaridin. Inward currents are shown as downward deflections. Odorant concentrations were plotted on a logarithmic scale. Each point represents the mean current response; error bars are s.e.m. n = 5–6 oocytes for each treatment. Treatments with high IR3535 concentrations (10^−4^ M, 10^−3^ M and 10^−2^ M) and Picaridin (10^−3^ M and 10^−2^ M) differed significantly from the no-repellent controls (two-way ANOVA, Bonferroni posttests, **: *P*<0.01; ***: *P*<0.001). Vertical and horizontal scale bars represent 100 nA and 1 min, respectively.

AaOR2 and AaOR8 were exposed to a range of IR3535 and Picaridin concentrations (10^−7^ M−10^−2^ M) in the presence of their respective odorants indole and octenol (both at 10^−7^ M) (Fig. 5A and 5C). Both compounds strongly and significantly reduced AaOR2 and AaOR8 responses to indole and octenol (*P*<0.01; Bonferroni posttest) in a concentration-dependent manner (Fig. 5B and 5D). IR3535 had a 4-fold stronger inhibitory effect on AaOR8 compared to AaOR2 based on extrapolated IC_50_ values (Fig. 1 and S5). Picaridin had statistically similar effects (IC_50_ values) on both receptors (Fig. 1 and S5). The inhibitory effects of IR3535 and Picaridin on AaOR2 and AaOR8 were reversible using a final exposure of 10^−7^ M indole or octenol, respectively (Fig. 5A and 5C).

## Discussion

We studied the actions of insect repellents DEET, 2-U, IR3535 and Picaridin on the activities of two *Aedes aegypti* ORs, AaOR2 and AaOR8, in the absence and presence of odorants specific to these ORs, indole (OR2) and octenol (OR8). In all cases, the ORs were expressed in *Xenopus* oocytes along with their common obligate co-receptor AaOR7. In the absence of odorant, DEET activated AaOR2 but not AaOR8, while 2-U activated AaOR8 but not AaOR2; neither receptor was activated by IR3535 or Picaridin. In the presence of odor, DEET strongly inhibited odorant-induced responses of AaOR8 but only slightly inhibited AaOR2, while 2-U strongly inhibited odorant-induced responses of AaOR2 but only slightly inhibited AaOR8; both receptors were equally and strongly inhibited by IR3535 or Picaridin. The observed OR activation by DEET and 2-U is consistent with previous physiological reports of adult OSNs and a molecular study of a larval OR. DEET alone activated two OSNs in the short blunt tipped sensilla (A-2) of *Ae. aegypti* [7]. 2-U acted as a mosquito attractant [22] and activated mosquito OSNs including an OSN sensitive to octenol [23], [36]. DEET alone also activated a larval OR and affected larval behavior in *An. gambiae* [3].

The dual activities of DEET and 2-U, activation and inhibition under different conditions, is consistent with the idea that these repellents may act on independent sites on the ORs. The activation properties of DEET and 2-U are consistent with the structural similarities to indole and octenol, respectively (Fig. 1), and suggest that these repellents may bind to and activate the odor binding site, albeit at lower affinity than the odorant. DEET and indole share an aromatic ring and a nitrogen-linked function. 2-U and octenol share a similar carbon backbone, and 2-U has a ketone group similar to the octenol analog 1-octen-3-one which was previously reported to activate AaOR8 [33]. Orthosteric modulation by DEET and 2-U is consistent with their structural similarities with their cognate ligands and with our data.

In our study, millimolar doses of repellents were necessary to achieve both odorant-independent activation and odorant-dependent inhibition of ORs. These high concentrations are consistent with the high amounts of repellents required in commercial formulations and the large quantities needed to elicit physiological responses in mosquito OSNs [4], [5], [37]. While the amount of repellents going into vapor phase is unknown, it is clear that large quantities are required to achieve close range protection against arthropod bites. At the physiological level, indole-sensitive neurons were activated by DEET only at high concentrations (apparent threshold of 100 µg) [37], while a dose-response curve revealed a 6 spike/s frequency increase over a 10,000 fold DEET increase (0.1 µg–1000 µg). A “DEET sensitive” OSN in short trichoid sensilla of *Cx. quinquefasciatus* was also activated by high concentrations (10 µg) of DEET [5].

Our observations have in common with prior studies that DEET activates OSNs at extremely high concentrations considering the reported sensitivity of these neurons to their cognate ligands. For example, in our study, both DEET and 2-U respectively activate OR2 and OR8 at 10^−3^ M while their respective ligands are active at 10^−8^ M (Fig. S1 and [33]), or 5 log steps higher sensitivity to the cognate ligand. Similarly, the detection threshold for the well-characterized octenol receptor located on the maxillary palps of *Cx. quinquefasciatus* is 0.1 ng [38] while 10,000 ng of DEET (5 log steps difference) activates an antennal OSN in the same insect [5]. Although it should be pointed out that based on differences in retention times observed in gas chromatography, DEET is less volatile than octenol.

We report the inhibitory (antagonist) property of all 4 repellents through negative modulation of odorant-induced OR activation. IR3535 and Picaridin alone failed to activate either receptor, but rather inhibited the receptor complex (OR2+OR7, OR8+OR7) regardless of the odor-binding subunit; AaOR2 and AaOR8 are highly divergent, sharing only 14% sequence identity. The common action of IR3535 and Picaridin on these otherwise divergent ORs suggests they target the common AaOR7 component. DEET and 2-U differentially inhibited odorant-induced activity of AaOR8 and AaOR2, but in opposite relationship with repellent induced activation, suggesting independent OR binding sites for activation and inhibition. The differential inhibitory activities of DEET and 2-U on AaOR2 and AaOR8 suggest that inhibitory binding sites for these repellents associate with the OR2 and OR8 subunits. Inhibitory activities of DEET and 2-U may thus be more influenced by differences in OR2 and OR8 sequence than the activities of IR3535 and Picaridin.

DEET alone has been shown to inhibit several classes of OSNs in insects, including lactic acid-sensitive OSNs in *Ae. aegypti* [19], [20], various *Drosophila* OSNs and the 1-octen-3-ol receptor neuron of *An. gambiae* [4]. Ditzen et al. [4] showed that responses of AgOR2 to 2-methylphenol and AgOR8 to racemic 1-octen-3-ol were differentially inhibited by DEET in the oocyte system with the strongest inhibitory effect on the latter.

The odor-inhibition activity of all four repellents was effective only at very high repellent concentrations (above 10^−4^ M). This is consistent with the high concentrations of repellents used in commercial formulations (e.g. DEET concentration ranges from 4 and 100% in commercial products). The inhibitory effects of all four repellents were reversible upon fresh exposure to the odorant alone, suggesting that the interaction between the inhibitors and the ORs is as labile as the one between the receptor and its cognate odorant.

It was previously suggested that DEET reduces OSN activity to experimentally applied airborne odorants through interactions between DEET and odor molecule in the release substrate in stimulus cartridges [5]. We did not measure whether DEET and octenol can form a stable complex in water that might mask the concentration of odor available for binding at high DEET concentrations. However, we did demonstrate that DEET does not chemically alter the structure of octenol (Fig. 4). We also showed that the solvent used for odor introduction, DMSO, had no measurable effect on OR activity when presented in the absence of odor molecules.

DEET, IR3535 and Picaridin all possess an amide moiety. Small amide derivatives have been shown to affect a wide range of molecular pathways through allosteric regulation of various proteins including proteases [39], [40], the cannabinoid receptor 1 (CB1) [41], the α7 nicotinic acetylcholine [42] and GABA_A_ receptors [43]. The broad activity of such compounds is mirrored by DEET's inhibitory effects on phylogenetically unrelated cation channels [4] and underscores that there might be alternative modes of action yet unknown.

Our results reconcile seemingly contradictory theories of DEET's mode of action. Previous studies suggested DEET decreases the sensitivity of OSNs to known attractants [20] and ORs [4] to their cognate odorants, or stimulates specific OSNs [5], [7] and ORs [3] that induce repellent behavioral responses. The excitatory and inhibitory properties of DEET and 2-U, as well as the non-specific inhibitory effects of IR3535 and Picaridin on ORs observed in our current study, support a model in which repellent-targeted OSNs elicit altered patterns of glomerular activity resulting in the scrambling of cognitive olfactory inputs and ultimately behavioral disruption.

## Materials and Methods

### Heterologous Expression of *AaOr2*, *AaOr7* and *AaOr8* in *Xenopus laevis* Oocytes

*AaOr2*, *AaOr7* and *AaOr8* cRNAs were synthesized from linearized pSP64DV expression vectors (Dr. L. J. Zwiebel, Vanderbilt University) using the mMESSAGE mMACHINE SP6 kit (Ambion). Following mechanical disruption of the *Xenopus laevis* ovaries, stage V-VI oocytes were treated for 30 min at room temperature under 70 rpm shaking with a 2 mg/mL collagenase (SIGMA, C6895) solution in Ca^2+^ free Ringer's buffer (96 mM NaCl, 2 mM KCl, 5 mM MgCl_2_ and 5 mM HEPES [pH 7.6]). All procedures were performed in accordance with the NIH Institutional Animal Care and Use Committee and NIH guidelines. Oocytes were subsequently washed 5 times with Ca^2+^ free Ringer's buffer, 5 times with Ca^2+^ free Ringer's buffer supplemented with 50 mg/mL gentamycin and 5 times with Ringer's buffer (96 mM NaCl, 2 mM KCl, 5 mM MgCl_2_/6H_2_O, 5 mM HEPES and 0.8 mM CaCl_2_ [pH 7.6]) supplemented with 5% heat inactivated horse serum, 50 mg/mL tetracycline, 100 mg/mL streptomycin and 550 mg/mL sodium pyruvate. Individual oocytes were allowed to recover overnight prior to injection with 10 ng of each cRNA and were recorded 4 to 6 days post-injection.

### Electrophysiological Recordings

Whole-cell currents were recorded using the two-microelectrode voltage clamp technique [44], [45]. Odorants and insect repellents were dissolved in 1% dimethyl sulfoxide (DMSO) final concentration. Prior to recording, stock solutions were diluted in Ringer's solution [pH 7.6] (96 mM NaCl, 2 mM KCl, 5 mM MgCl_2_, 5 mM HEPES and 0.8 mM CaCl_2_) to the indicated concentrations before being applied to *Xenopus* oocytes in a RC-3Z oocyte recording chamber (Warner Instruments) connected to a manual gravity perfusion system. Oocytes were continuously perfused by either pure Ringer's solution or exposed for 8 sec to 10^−7^ M of the odorant alone, 10^−7^ M of the repellent alone, or to solutions of 10^−7^ M odorant in combination with sequentially fixed increasing concentrations of inhibitors, all dissolved in Ringer's solution. An 8 sec stimulation was chosen to stay consistent with other functional OR studies using similar odorant delivery systems [28]. To avoid residual repellent effects, each oocyte was exposed to only one of the four tested repellents. Odorant-induced currents were recorded with an OC-725C oocyte clamp (Warner Instruments) at a holding potential of −80 mV. Between stimulations, oocytes were allowed to return to their membrane resting potential by washing out the odorants or the odorant and inhibitor using pure Ringer's solution. The recovery time was defined as the time required for agonist-induced responses to abate and to reach levels identical to pre-stimulation levels. Data acquisition and analysis were carried out with Digidata 1440A and pCLAMP10 software (Axon Instruments).

### Data Analysis

For the desensitization analysis (GraphPad Prism5 Software, Inc.), the perfusion system consisted of a unique stimulus source for the repeated administration of the agonist. Normalization of the current responses for AaOR2+AaOR7 and AaOR8+AaOR7 was performed by calculating the ratio of a given response to the current elicited by the the first exposure defined as 100% based on ratio defined by equation [1] (Fig. S6). Linear regression was performed using Prism5.

In subsequent experiments, the perfusion system required additional delivery sources for the application of serial dilutions of inhibitors (Fig. S6). Data normalization was performed by calculating the ratio of a given response to the average response elicited by the ligand alone (first and last stimulation) (Fig. S6).

Statistical analyses of the logIC_50_ means were performed using an ordinary one-way analysis of variance (ANOVA) followed by a Tukey Kramer multiple comparison post-test. Results with *P*<0.05 were considered statistically significant. In all figures, graphical results are shown as means and standard error of the mean of five or more independent oocytes. IC_50_ values for individual compounds were extrapolated using the non-linear regression curve fit function provided in Prism5.

### Chemical Analyses

Organic chemicals were extracted from physiological saline solutions containing 1% dimethyl sulfoxide (DMSO) and (*R*)-(−)-1-octen-3-ol at 10^−5^ M or 1% DMSO and (*R*)-(−)-1-octen-3-ol at 10^−5^ M and N,N-diethyl-3-methylbenzamide (DEET) at 10^−3^ M using ethyl acetate. One milliliter of the experimental solution was shaken then vortexed with 500 µL of ethyl acetate. After the ethyl acetate separated from the physiological saline solution, 300 µL of this supernatant was transferred into a cone vial for analysis. A one microliter aliquot of this supernatant was injected into an Agilent 6890 gas chromatograph (GC) equipped with a HP-5 capillary column (cross-linked 5% PH ME Siloxane; film thickness 0.25 µm; length 30 m; internal diameter 0.25 mm) and flame ionization detector. After an initial temperature of 50°C held for 2 min following sample injection, the temperature of the GC oven was increased 15°C/min to 235°C which was held for 8 min. Identifications of peaks in the gas chromatograms were verified using an Agilent 7890A GC coupled with an Agilent 5975C mass spectrometer (MS) also equipped with an HP-5 capillary column as previously described. The temperature program used for GC/MS analysis was identical to the regime used in GC studies. Authentic spectra for DMSO, octenol and DEET from the NIST (National Institute of Standards) reference library of mass spectra were matched to mass spectra obtained from our samples. Five replicates for each experimental solution were conducted. For each replicate, the areas of GC peaks for DMSO, octenol and DEET were calculated using GC/EAD software from Syntech, The Netherlands. Means for DMSO and (*R*)-(−)-1-octen-3-ol obtained for solutions with or without DEET were compared using a *t*-test.

### Chemicals

Indole (99+%) was obtained from Aldrich Chemical Co., Milwaukee, WI, USA. (*R*)-(−)-1-octen-3-ol [99.6% (*R*) form] was custom synthesized by Bedoukian Research, Inc. The repellents used in this study, their purity and source were: DEET N,N-diethyl-3-methylbenzamide (99.2%) and 2-undecanone (99%), Aldrich Chemical Co., Milwaukee, WI, USA; IR3535 3-[N-butyl-N-acetyl]-aminopropionic acid ethyl ester (>95%), Merck, Rahway, NJ, USA and Picaridin 2-(2-hydroxyethyl)-1-piperidine carboxylic acid 1-methylpropyl ester (>95%), Bayer, USA.

## Supporting Information

Figure S1 AaOR2+AaOR7 dose-response curve to indole. Concentration-response plots of AaOR2+AaOR7 to indole. Odorant concentrations were plotted on a logarithmic scale. Each point represents the mean current response; vertical bars are s.e.m. (n = 5 oocytes).
(0.29 MB TIF)

Figure S2 Desensitization of AaORs by odorants. Activation of AaOR2+AaOR7 and AaOR8+AaOR7 by repeated exposures of 10−7 M indole and 10−7 M octenol [(R)-(−)-1-octen3-ol], respectively. (A) Response traces of AaOR2+AaOR7 and AaOR8+AaOR7 are recorded in nano-ampere (nA). Inward currents are shown as downward deflections. Vertical and horizontal scale bars represents 100 nA and 1 min, respectively. (B) Fractional activities (left Y-axis) are expressed as percentages with respect to the initial exposure defined as 100%. The data points were fitted using a linear regression model (solid lines): AaOR2 (r2 = 0.94, slope = −2.738±0.2781, n = 7); AaOR8 (r2 = 0.96, slope = −3.930±0.2965, n = 10). The two slopes were significantly different (P<0.05, Student's t-test). Histogram of the time intervals (right Y-axis) between stimulations of AaOR2+AaOR7 and AaOR8+AaOR7 by serial exposures of 10−7 M indole and 10−7 M octenol, respectively. Each point represents the mean and vertical error bars indicate s.e.m. Mean time intervals were not statistically different (two-way ANOVA, Bonferroni posttests, P>0.05).
(0.56 MB TIF)

Figure S3 Insect repellents do not elicit currents in water-injected oocytes (control). Water-injected oocytes did not display currents following exposure to increasing concentrations of DEET, 2-undecanone (2-U), IR3535 or Picaridin in the presence of 10−7 M octenol [(R)-(−)-1-octen3-ol] or 10−7 M indole (n = 5).
(0.81 MB TIF)

Figure S4 High concentration of 2-Undecanone prolongs AaOR8+AaOR7 recovery. Recovery times of the AaOR2+AaOR7 (AaOR2) and AaOR8+AaOR7 (AaOR8) complexes following 10−2 M exposure to IR3535, Picaridin, DEET, 2-undecanone (2-U) or to agonist alone. Bars represent the mean recovery time; error bars are s.e.m; n = 5–6 oocytes for each treatment. Bar labeled with three asterisks indicates P<0.0001 (ANOVA test with Tukey posttest).
(0.59 MB TIF)

Figure S5 Relative effectiveness of IR3535, Picaridin, DEET and 2-undecanone on AaOR2+AaOR7 and AaOR8+AaOR7 responses. Half maximal inhibitory concentration (IC50) ranking profile of IR3535, Picaridin, DEET and 2-undecanone (2-U) on AaOR2+AaOR7 and AaOR8+AaOR7. ns, not significant; *, P<0.05; **, P<0.01 and ***, P<0.001 (ANOVA test with Tukey post test). Odorant concentrations were plotted on a logarithmic scale. Each point represents the mean and error bars indicate s.e.m. n = 5 oocytes for each treatment.
(0.94 MB TIF)

Figure S6 Gravity-driven perfusion system and normalization method. Each of the 6 fractional activities was calculated by measuring each current (Yn) elicited by the odorant in the presence of one of six doses (10−7 M to 10−2 M) of repellents divided by the average of the sum of the initial (X0) and final (X1) ligand-evoked currents as shown in the equation.
(1.35 MB TIF)
