# Supplementary figures and images for: Insect Repellents: Modulators of Mosquito Odorant Receptor Activity
Source: PLoS One. 2010 Aug 11;5(8):e12138. doi: 10.1371/journal.pone.0012138 (PMC2920324; doi:10.1371/journal.pone.0012138)

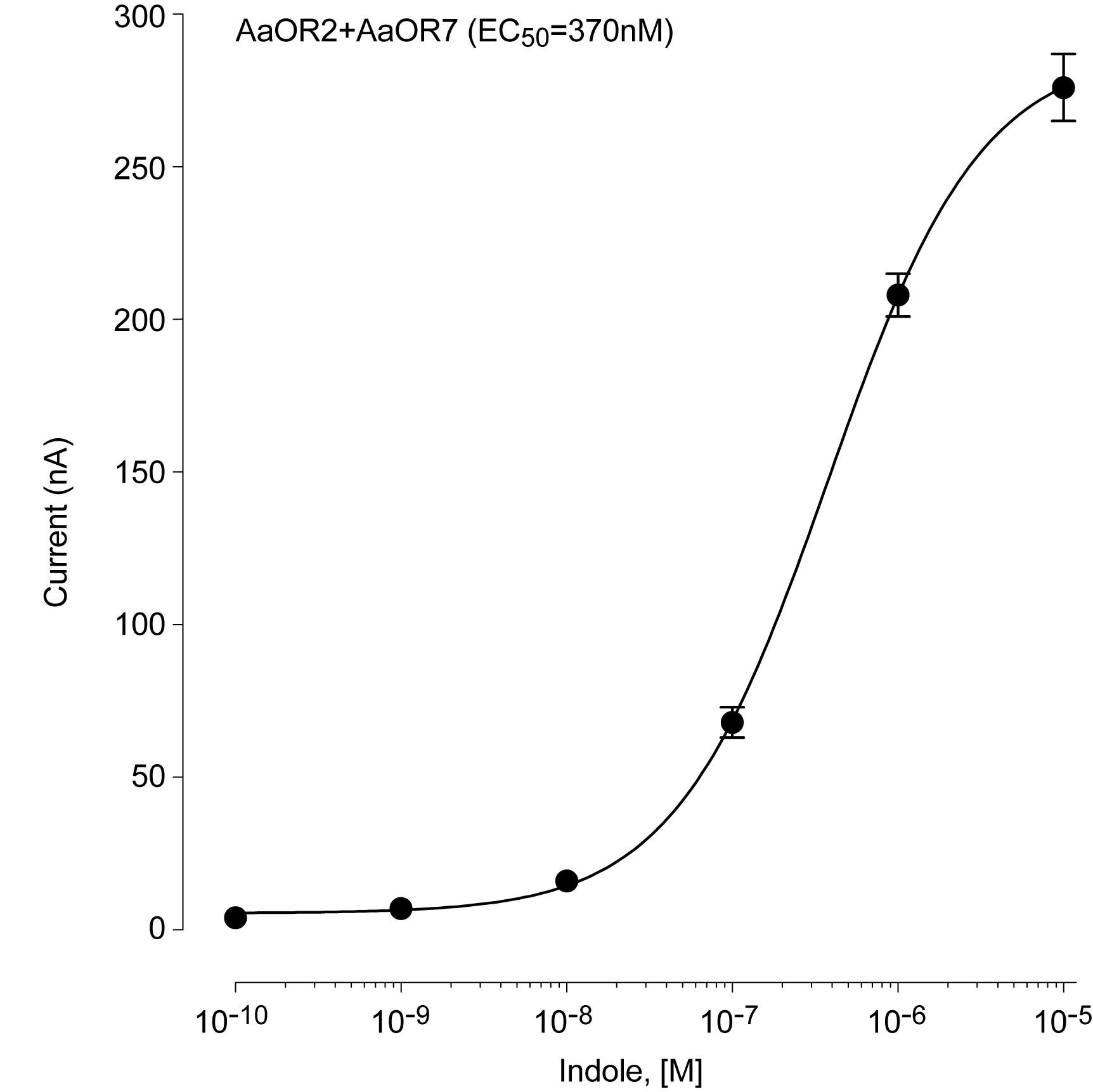

Supplement: Figure S1 — AaOR2+AaOR7 dose-response curve to indole. Concentration-response plots of AaOR2+AaOR7 to indole. Odorant concentrations were plotted on a logarithmic scale. Each point represents the mean current response; vertical bars are s.e.m. (n = 5 oocytes). (0.29 MB TIF) [file pone.0012138.s001.tif]

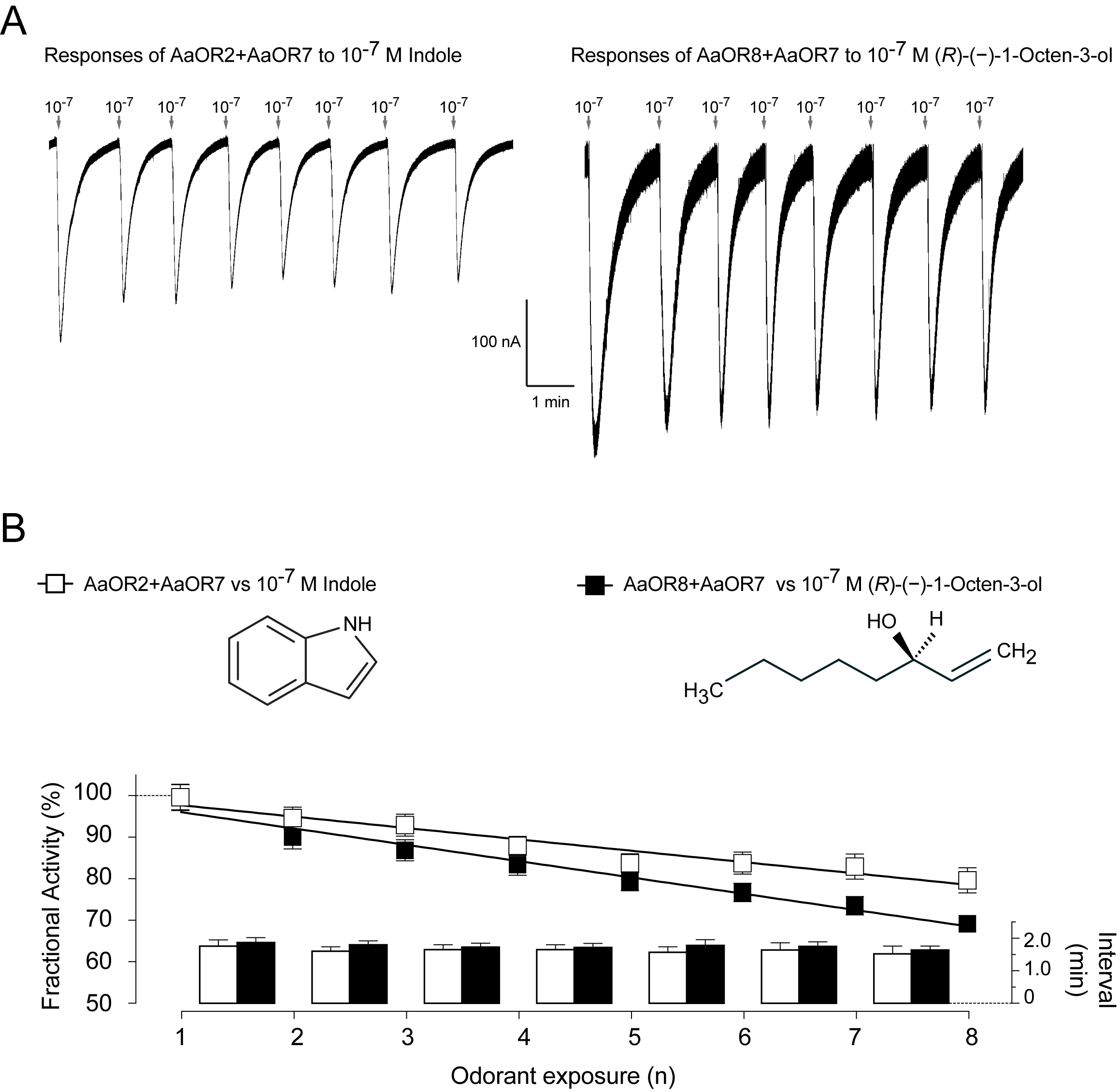

Supplement: Figure S2 — Desensitization of AaORs by odorants. Activation of AaOR2+AaOR7 and AaOR8+AaOR7 by repeated exposures of 10−7 M indole and 10−7 M octenol [(R)-(−)-1-octen3-ol], respectively. (A) Response traces of AaOR2+AaOR7 and AaOR8+AaOR7 are recorded in nano-ampere (nA). Inward currents are shown as downward deflections. Vertical and horizontal scale bars represents 100 nA and 1 min, respectively. (B) Fractional activities (left Y-axis) are expressed as percentages with respect to the initial exposure defined as 100%. The data points were fitted using a linear regression model (solid lines): AaOR2 (r2 = 0.94, slope = −2.738±0.2781, n = 7); AaOR8 (r2 = 0.96, slope = −3.930±0.2965, n = 10). The two slopes were significantly different (P<0.05, Student's t-test). Histogram of the time intervals (right Y-axis) between stimulations of AaOR2+AaOR7 and AaOR8+AaOR7 by serial exposures of 10−7 M indole and 10−7 M octenol, respectively. Each point represents the mean and vertical error bars indicate s.e.m. Mean time intervals were not statistically different (two-way ANOVA, Bonferroni posttests, P>0.05). (0.56 MB TIF) [file pone.0012138.s002.tif]

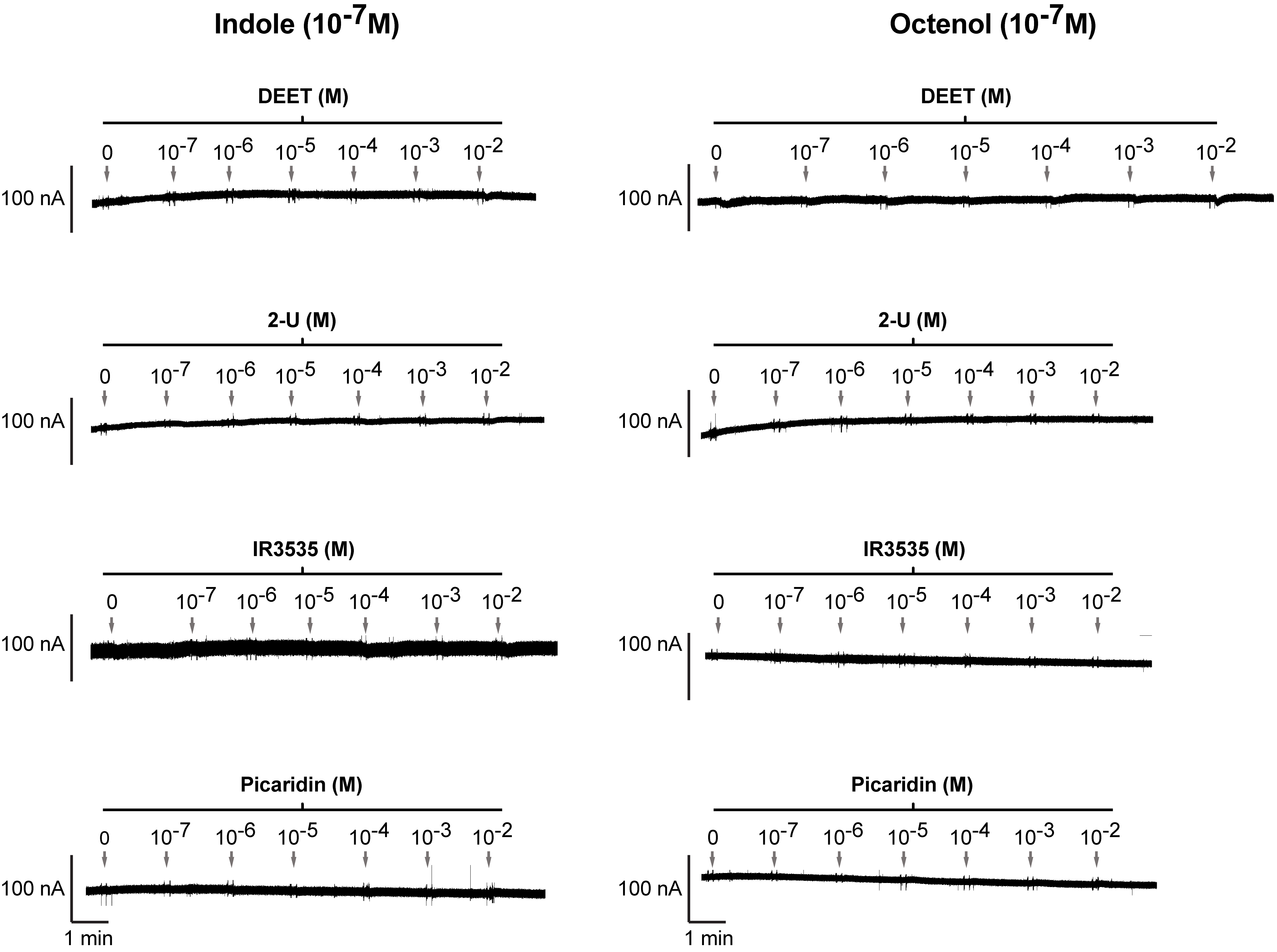

Supplement: Figure S3 — Insect repellents do not elicit currents in water-injected oocytes (control). Water-injected oocytes did not display currents following exposure to increasing concentrations of DEET, 2-undecanone (2-U), IR3535 or Picaridin in the presence of 10−7 M octenol [(R)-(−)-1-octen3-ol] or 10−7 M indole (n = 5). (0.81 MB TIF) [file pone.0012138.s003.tif]

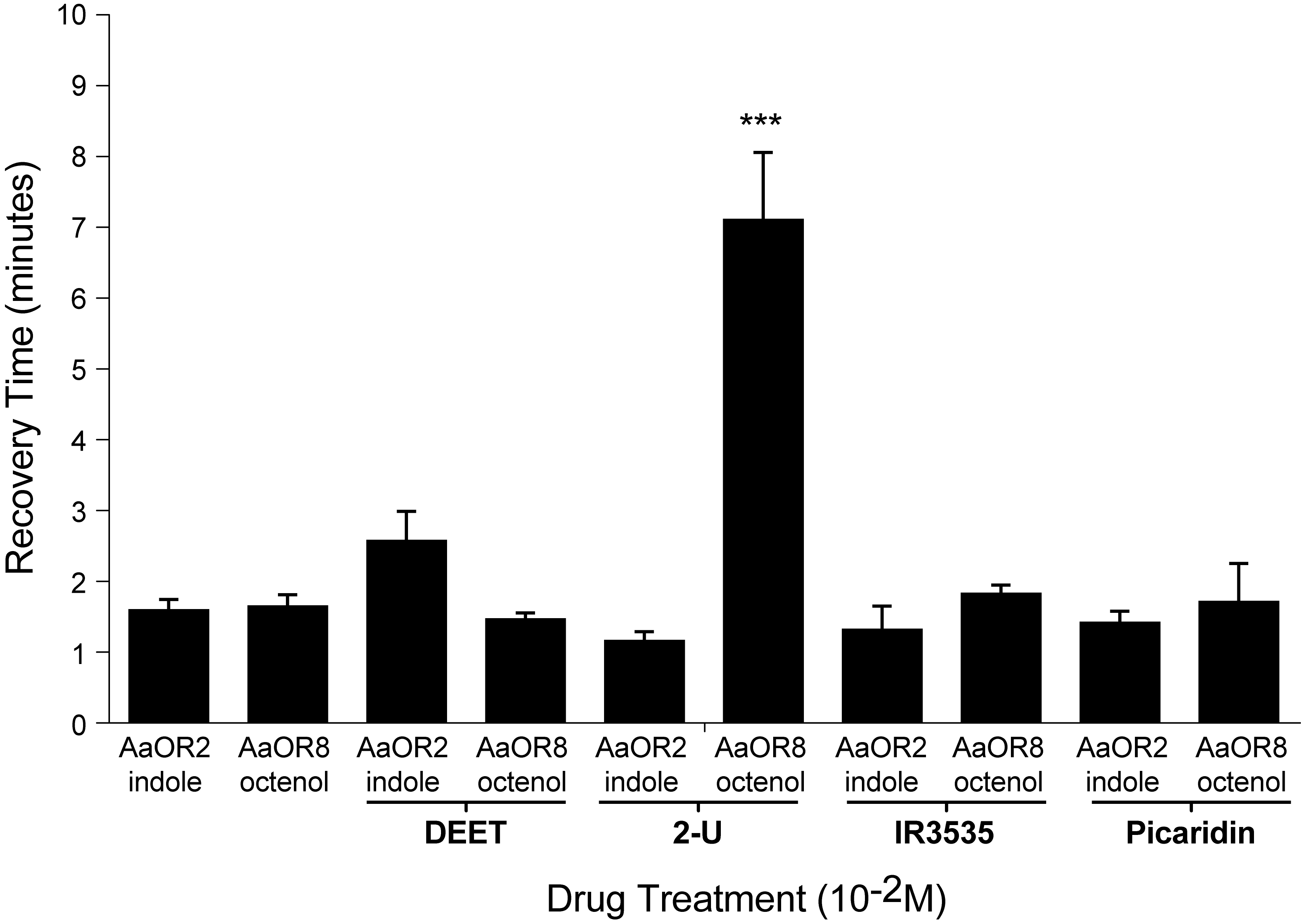

Supplement: Figure S4 — High concentration of 2-Undecanone prolongs AaOR8+AaOR7 recovery. Recovery times of the AaOR2+AaOR7 (AaOR2) and AaOR8+AaOR7 (AaOR8) complexes following 10−2 M exposure to IR3535, Picaridin, DEET, 2-undecanone (2-U) or to agonist alone. Bars represent the mean recovery time; error bars are s.e.m; n = 5–6 oocytes for each treatment. Bar labeled with three asterisks indicates P<0.0001 (ANOVA test with Tukey posttest). (0.59 MB TIF) [file pone.0012138.s004.tif]

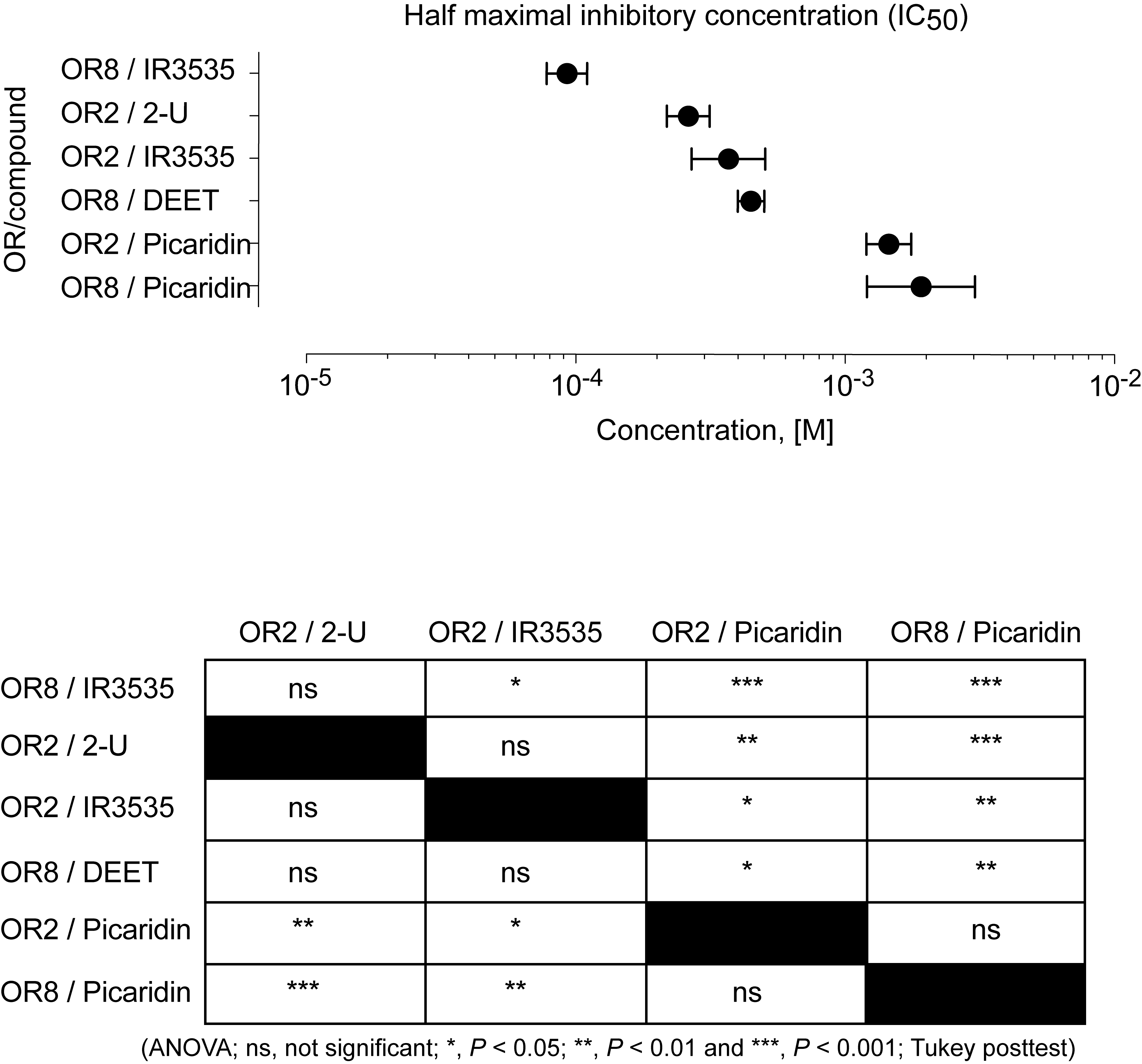

Supplement: Figure S5 — Relative effectiveness of IR3535, Picaridin, DEET and 2-undecanone on AaOR2+AaOR7 and AaOR8+AaOR7 responses. Half maximal inhibitory concentration (IC50) ranking profile of IR3535, Picaridin, DEET and 2-undecanone (2-U) on AaOR2+AaOR7 and AaOR8+AaOR7. ns, not significant; *, P<0.05; **, P<0.01 and ***, P<0.001 (ANOVA test with Tukey post test). Odorant concentrations were plotted on a logarithmic scale. Each point represents the mean and error bars indicate s.e.m. n = 5 oocytes for each treatment. (0.94 MB TIF) [file pone.0012138.s005.tif]

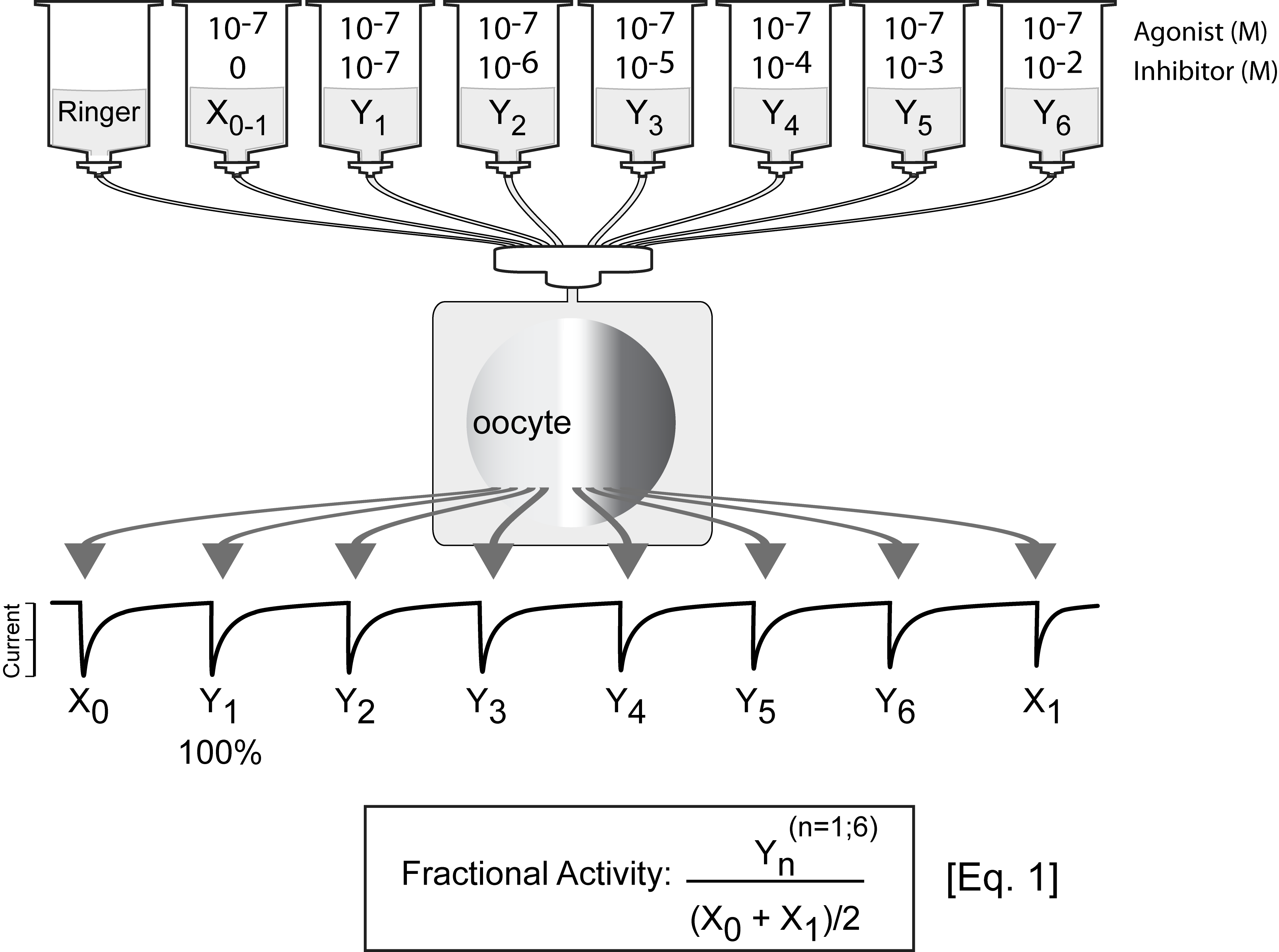

Supplement: Figure S6 — Gravity-driven perfusion system and normalization method. Each of the 6 fractional activities was calculated by measuring each current (Yn) elicited by the odorant in the presence of one of six doses (10−7 M to 10−2 M) of repellents divided by the average of the sum of the initial (X0) and final (X1) ligand-evoked currents as shown in the equation. (1.35 MB TIF) [file pone.0012138.s006.tif]
